# Binocular Vision Anomalies in Scotland at Age 3.5–5.5 Years: An Epidemiological Study

**DOI:** 10.1007/s44402-026-00042-2

**Published:** 2026-03-30

**Authors:** Bruce J. W. Evans, Lee Pentland, Benjamin E. W. Evans, David F. Edgar, Rakhee Shah, Miriam L. Conway

**Affiliations:** 1 Department of Optometry and Visual Sciences, City St George’s, University of London, London, UK; 2 Ninewells Hospital, NHS Tayside, Dundee, UK

**Keywords:** Binocular vision anomalies/dysfunction, Children, Heterophoria, Prevalence, Strabismus, Vision screening

## Abstract

**Purpose:**

Scotland has comprehensive vision screening at age 3.5–5.5 years, with ~85% participation (40,000–50,000 episodes annually). Orthoptists deliver the screening, including presenting vision and tests for binocular vision anomalies (BVAnom). The aims were to investigate (1) changes in the prevalence of BVAnom, 2013–2022 and (2) whether less comprehensive screening of solely presenting vision would detect BVAnom.

**Methods:**

Data from eight Scottish Health Boards were available for 2013–2014, 2014–2015, 2015–2016, 2020–2021 and 2021–2022. Binocular vision tests included cover test, ocular motility (OM) and additional tests (near point of convergence, 20Δ base out, pass/fail stereopsis). Data were analysed to determine the prevalence of various BVAnom and adequacy of screening if based solely on vision.

**Results:**

From 2013 to 2022, there was a statistically significant increase in prevalence of exotropia (including intermittent; *r*^2^ = 0.983, *p* = 0.001) and of any strabismus (including intermittent; *r*^2^ = 0.887, *p* = 0.02), with strabismus prevalence ~2% in 2020–2022. Prevalence of OM anomalies remained stable (*r*^2^ = 0.364, *p* = 0.28). The prevalence of BVAnom for each year studied, consecutively, was 3.02, 3.78, 3.83, 4.87, 4.89% (*r*^2^ = 0.930, *p* = 0.008). If vision screening had been confined to presenting vision, 342–512 cases of BVAnom would have been missed each year, increasing over time (*r*^2^ = 0.934, *p* = 0.007).

**Conclusions:**

In a large population of children in Scotland aged 3.5–5.5 years, the prevalence of BVAnom is increasing, especially exotropia. Many cases of BVAnom would not be detected by solely assessing presenting vision, highlighting the benefits of including binocular vision tests in vision screening. It is recommended that vision screening is repeated during the school years.

Key Points

- In a large population of children in Scotland aged 3.5–5.5 years, the prevalence of binocular vision anomalies is increasing.
- As might be expected from increasing myopia, exotropia is becoming more prevalent, but there has been no commensurate decrease in the prevalence of eso-deviations.
- Many cases of binocular vision anomalies would not be detected by solely assessing presenting vision/visual acuity, highlighting the benefits of including binocular vision tests in vision screening.

## Introduction

Children’s vision screening in the UK normally occurs at age 4–5 years [1]. This approach [2] is based on a literature review [3] that assumes the purpose of vision screening is to address amblyopia, and which does not consider other conditions or amblyopia risk factors [3].

A study [4] noted that the UK National Screening Committee (NSC) recommendation [5] is unusual in only screening children’s vision once. In 2015, of the 25 EU countries with a vision screening programme, 71% screened more than once [6], which has also been recommended in North America [7]. There is a higher risk of failing vision screening in families receiving state benefits [8].

A 2021 systematic review argued that the UK vision screening programme is preferable for detecting amblyopia when compared with autorefraction or photorefraction at a younger age [9]. Often, publications on vision screening still focus on amblyopia detection [10], although a study of 3721 children in the UK found that 42% of those who failed vision screening had manifest strabismus [11]. Manifest strabismus significantly affects quality of life [12], dating [13] and employment [14] while correction improves quality of life [15–17], and strabismus prognosis is improved by early intervention [18].

McCullough and Saunders investigated child vision screening based on the UK NSC protocol with 294 children aged 4–5 years [19]. They found moderate sensitivity (70.4%) and specificity (82.2%) for detecting strabismus and/or significant refractive error.

Since 2013, children in Scotland registered with a General Medical Practitioner and not already in the care of the Hospital Eye Service (HES) have been invited to vision screening by orthoptists in the See4School programme [20]. Approximately 55,000 children, aged 3.5–5.5 years, are eligible annually. Fully anonymised data are collated for basic audit by the Scottish Health Boards (HBs) and descriptions of these data have been published, including overall performance of the programme [20, 21] and refractive error data [22]. Refractive errors were determined for children who, on failing vision screening, were referred for eye examinations (~5000 per annum). In contrast, the present manuscript reports the results of testing for binocular vision anomalies (BVAnom), attempted in all children attending screening (~39,000–48,000 per annum), using the following tests: cover test (distance and near), ocular motility (OM), near point of convergence, response to 20Δ base out test (Prism Reflex Test, PRT) and pass/fail stereopsis based on the first plate of the Frisby stereo test (Frisby Stereotest; frisbystereotest.com) or gross plates of the TNO test (Lameris Group; lameris-group.nl) [20].

The prevalence of BVAnom shows considerable variability across regions, populations and ages [23]. For example, the prevalence of strabismus varies from 0.11 to 8.7% [23]. Prevalence data for young children in the UK are limited [18]. The first aim of this retrospective epidemiological study is to determine the prevalence of BVAnom in children aged 3.5–5.5 years in Scotland, quantifying changes from academic years 2013–2022. The second aim is to investigate the extent to which a less comprehensive screening programme of presenting vision alone fails to detect BVAnom.

In this paper, the term strabismus is used to include all cases where a manifest strabismus was recorded at any point during cover testing (i.e., including intermittent strabismus). The term deviation was generically used to describe both strabismus and heterophoria (e.g., exo-deviation includes all cases of exophoria and exotropia) [18]. The term presenting vision is used to describe the orthoptist’s measurement of either unaided vision or, if the child presented to screening with spectacles, the measurement with spectacles [24].

## Methods

The research followed the tenets of the Declaration of Helsinki and proceeded after UK Health Research Authority and institutional approval and a data sharing agreement. The vision screening methods are described elsewhere [21].

Author LP, who is Lead for Child Vision Screening in Tayside and co-ordinates the audit of vision screening data in Scotland, merged data from different HBs and provided deidentified data of those who failed screening and for whom data on BVAnom were available for the following academic years: 2013–2014, 2014–2015, 2015–2016, 2020–2021 and 2021–2022. These years were selected because national data collection was started in 2013–2014 and quality control checks were rigorously employed for the first 3 years, and again from 2020 to 2022. After data cleaning, the data were checked independently by two co-authors, and any discrepancies were resolved by discussion. The NHS Scotland Information Services Division (ISD) provided, for each year, the total number of children eligible for and invited to attend screening, and the number who attended screening.

The criteria for failing vision screening were any of the following:

1. Abnormal monocular presenting vision (criteria in Pentland and Conway [21]);
2. Presenting vision interocular difference (IOD) of three letters or more;
3. Manifest or intermittent strabismus on cover testing;
4. Heterophoria that is not well-compensated. This is likely to include any hyperphoria; any esophoria or exophoria that is poorly controlled (described below as clinically significant exophoria) as indicated during the cover test, or failed stereoacuity (failed first plate of Frisby or gross plates of TNO tests);
5. Abnormal near point of convergence in combination with abnormal PRT (in the orthoptist’s opinion, convergence reduced sufficiently to be likely to cause problems requiring referral);
6. OM defects: incomitance, including pattern strabismus (e.g., V-pattern) that the orthoptist judged likely to decompensate or cause symptoms, or was suspicious for pathology;
7. Other anomalies (e.g., nystagmus, ptosis, pupillary defects);
8. Unable to complete any of the above tests.

When the screening results were digitised, data on BVAnom were entered in three database fields. The first of these was the cover test results, coded as in the first two columns of Table 1. Cover testing was always attempted for near and distance vision, although the precise distance that was used for distance testing varied with different venues. The Table 1 coding descriptions that include ‘intermittent’ signify a strabismus that was present sometimes but not always during cover testing: intermittent at both distance and near, or constant strabismus for near vision but not distance vision (etc.). The cover test data were analysed according to the first- and second-level summary classifications in Table 1.

**Table 1:** Cover test codes used by orthoptist vision screeners (columns 1–2) and summary classifications in data analysis (columns 3–4) by researchers.

Code	Description	First-level summary classification	Second-level summary classification
1	No apparent deviation	No cover test abnormality	
2	Exophoria	Classified, from screener comments, as clinically significant exophoria/not (see later)	Heterophoria, exo-deviation
3	Esophoria	All classified as esophoria	Heterophoria, eso-deviation
4	Intermittent right esotropia	Intermittent esotropia	Strabismus, eso-deviation
5	Intermittent left esotropia		
6	Intermittent alternating esotropia		
7	Intermittent right exotropia	Intermittent exotropia	Strabismus, exo-deviation
8	Intermittent left exotropia		
9	Intermittent alternating exotropia		
10	Right esotropia	Constant esotropia	Strabismus, eso-deviation
11	Left esotropia		
12	Alternating esotropia		
13	Right exotropia	Constant exotropia	Strabismus, exo-deviation
14	Left exotropia		
15	Alternating exotropia		
16	Right hyperphoria	Vertical heterophoria	Heterophoria, vertical deviation
17	Left hyperphoria		
18	Alternating hyperphoria		
19	Right hypophoria		
20	Left hypophoria		
21	Alternating hypophoria		
22	Right hypertropia	Vertical strabismus	Strabismus, vertical deviation
23	Left hypertropia		
24	Alternating hypertropia		
25	Right hypotropia		
26	Left hypotropia		
27	Alternating hypotropia		
28	Other (free text in Comments field)	See ‘Results’	

The second database field about BVAnom was OM, coded as 0 for normal and 1 for abnormal. When an abnormality was recorded, typically the orthoptist entered a free-text description as ‘Comments’. These were inspected and classified [25].

Additional tests (near point of convergence, 20Δ base out, pass/fail stereopsis) were used by the orthoptist screeners to aid their decision-making [20], but the results were not formally recorded, other than sometimes in informal free-text comments.

### Statistical Analysis

The main analyses calculated the prevalence each year of various BVAnom [18]. Data were included for the eight HBs that provided a dataset for every year. For prevalence figures, the denominator is the number of children screened in these eight HBs. The policy for missing data was to make no imputations (see ‘Strengths and Limitations’ section of ‘Discussion’). Continuous variables were tested for normality, and parametric and non-parametric statistics were used as appropriate for each continuous variable.

Typically, screening starts at the end of August (nursery term starts mid-August) and ends in the summer of the following year, before the schools’ return. In the year directly affected by the COVID pandemic (2020–2021), screening started as usual in mid-August 2020, followed by a pause of ~2 ½ months. To make up for the delay, many HBs established screening clinics over the summer months (2021) in various locations (community centres, health centres, hospital clinics). In some HBs, the screening overran into the next screening year by up to 2 months (completion before the October 2021 mid-term break). Therefore, the mean age of screening was probably slightly older in the 2020–2021 year. The date of screening is not stored in the database, and therefore, this delay cannot be quantified. For the 2021–2022 year, the situation was back to normal.

## Results

**Fig. 1:**
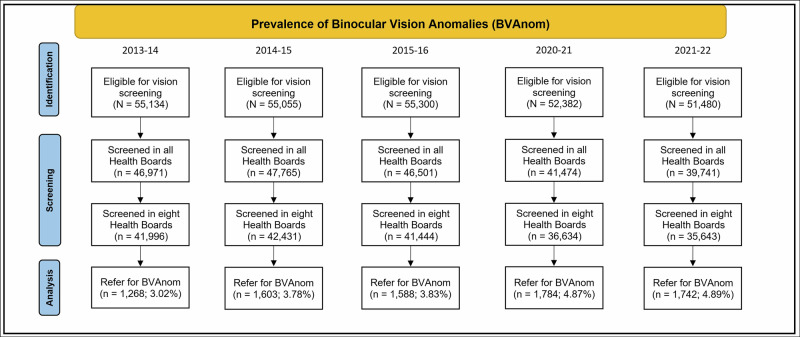
Preferred Reporting Items for Systematic Reviews and Meta-Analyses (PRISMA) flow diagram. For all years, the number who are eligible and attended for vision screening does not include children already under the Hospital Eye Services  . See below for more details on samples at each stage.

The Preferred Reporting Items for Systematic Reviews and Meta-Analyses (PRISMA) flow diagram (Fig. 1) illustrates the screening process. The numbers presented in the top two rows represent data for all 15 Scottish HBs provided by the ISD. The number of children eligible for screening excludes children already under the HES or whose parents/carers opted out of screening. The third and fourth rows refer specifically to children from the eight HBs that consistently submitted data across all 5 years (Ayrshire and Arran, Forth Valley, Greater Glasgow and Clyde, Grampian, Highland, Lanarkshire, Lothian, and Tayside). These eight were among the nine most populous HBs in Scotland, comprising over 85% of Scotland’s population according to 2021 population data [26].

### First Aim: Prevalence of BVAnom

#### Cover Test Data

Table 2 shows cover test results, using the first- and second-level summary classifications detailed in Table 1. The figures for constant esotropia include cases that are unilateral or alternating but do not include intermittent cases, which are listed separately, and similarly for exotropia. The cover test code of 28 (Table 1) was used for ‘Other (free text in Comments field)’ in only 76 records in the 5 years of data, excluding those who failed for a BVAnom because of an OM abnormality. For 36/76 cases, the Comment was that the test could not be completed, and for 14 cases, the comment was left blank. The remainder were idiosyncratic comments (e.g., ‘Mum requests appointment’) and were considered no further.

**Table 2:** Summary data for the prevalence of strabismus and heterophoria.

	2013–2014	2014–2015	2015–2016	2020–2021	2021–2022
Constant esotropia	0.60%	0.71%	0.54%	0.56%	0.72%
Intermittent esotropia	0.28%	0.29%	0.26%	0.38%	0.34%
All esotropia	0.88%	1.00%	0.80%	0.94%	1.05%
Constant exotropia	0.09%	0.14%	0.13%	0.15%	0.13%
Intermittent exotropia	0.43%	0.54%	0.55%	0.90%	0.98%
All exotropia	0.52%	0.68%	0.67%	1.05%	1.12%
Vertical strabismus	0.012%	0.014%	0.017%	0.018%	0.014%
Any strabismus^a^ (including intermittent)	1.42%	1.69%	1.49%	2.00%	2.19%
Vertical heterophoria^a^	0.036%	0.047%	0.034%	0.049%	0.059%
Esophoria^a^	1.06%	1.51%	1.82%	2.25%	2.22%
Clinically significant exophoria^a^	0.060%	0.108%	0.152%	0.076%	0.059%

^a^Cover test codings included in the overarching category of BVAnom (see later).

*BVAnom* binocular vision anomalies.

**Fig. 2:**
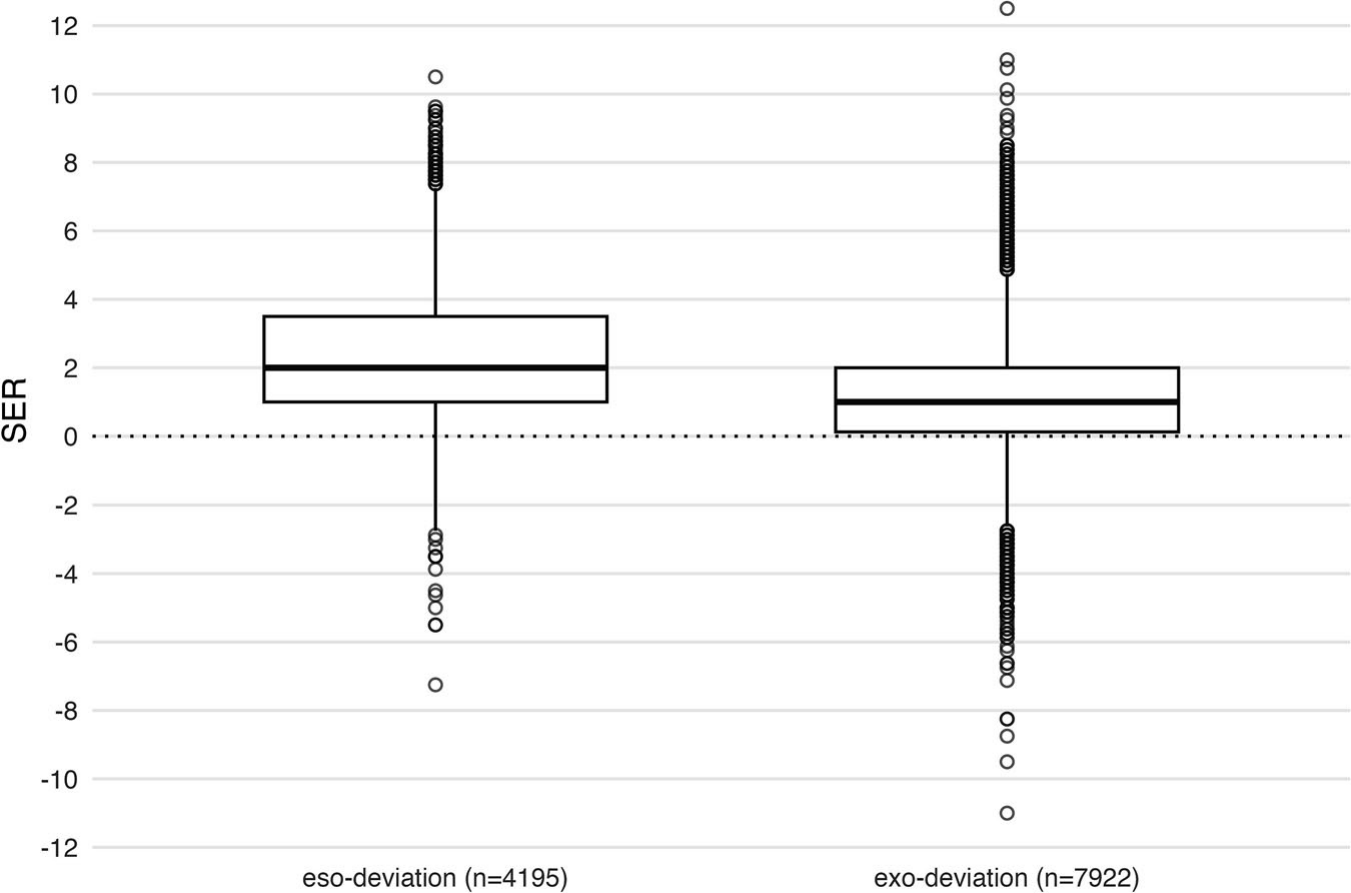
Boxplot illustrating refractive errors (right eye SER) in children with an eso-deviation compared with those with an exo-deviation. The horizontal line within each box is the median, and the upper and lower box limits represent the 75th and 25th percentiles, respectively. Bars outside the boxes represent 1.5× interquartile range, and circles are datapoints outside this range. SER spherical equivalent refraction.

The literature indicates an association between myopia and exo-deviations and hyperopia and eso-deviations [18]. This was investigated by pooling data for all 5 years in the eight HBs of all children with refractive error data [22] and a cover test code (Table 1) indicating eso-deviations or exo-deviations. The right eye spherical equivalent refraction (SER) in children with exo-deviations was significantly (Mann–Whitney *U* test, *p* < 0.001) less hyperopic (median: +1.00 D, IQR: +0.13 to +2.00 D; mean: +1.17 D) than in children with eso-deviations (median: +2.00 D, IQR: +1.00 to +3.50 D; mean: +2.42 D). A boxplot highlights more extreme outliers in the exo-deviations group (Fig. 2). In view of this finding and the increasing prevalence of myopia already reported in this dataset [22], it was hypothesised that there would be increasing exotropia. This is revealed in Table 2 and the trend for increasing prevalence of exotropia (including intermittent) over time is statistically significant (positive correlation, *r*^2^ = 0.983, *p* = 0.001). There was no complementary trend for all cases of esotropia to reduce over time (positive correlation, *r*^2^ = 0.293, *p* = 0.35). Considering any strabismus (constant and intermittent, horizontal and vertical), there was a statistically significant increase over time (positive correlation, *r*^2^ = 0.887, *p* = 0.02). The prevalence of vertical heterophoria (Table 2) was relatively stable over the period studied (*r*^2^ = 0.645; *p* = 0.10).

Over the period studied, there were 1452 cases with a cover test coding of exophoria and a comment. Of these, 220 had an OM coding of 1 (abnormal) and were considered no further because these cases would have required referral regardless of any comments about the exophoria. For the remaining 1232 cases, the comments were inspected to detect cases that were likely to have failed the screening because of concerns about the exophoria (see introduction). These were defined as clinically significant exophoria in Table 2.

#### Ocular Motility Data

The prevalence of an abnormal result at the OM test did not change significantly over the years (0.68, 0.53, 0.47, 0.95, 0.68%, consecutively; positive correlation, *r*^2^ = 0.364, *p* = 0.28). For cases with OM coded as abnormal with comments relating to OM, the comments were inspected and classified [25]. Where appropriate, descriptions received two classifications, and synonyms were classified appropriately (e.g., Browns, Browns bilateral, Browns L [left eye] were all classified as Brown’s syndrome). Descriptions of a muscle underaction were classified together with descriptions of underaction of the same muscle in combination with the pattern of muscle sequelae [25]. The results, for conditions encountered more than once across the 5 years, are summarised in Table 3. There were 12 further descriptions that each occurred only once and were classified following the principles in Table 3. Owing to the rarity of most of the conditions and categories in Table 3, the numbers are too low for meaningful comparisons between years.

**Table 3:** Results of classification of free-text comments in order of frequency of occurrence, number over the 5 years of data and prevalence (shown as percentage, Prev. and as the number that would need to be seen for one case to occur) for each description and classification (when first mentioned in the table) from the total sample of *N* = 198,148.

Description	*n*	Prev.	Rate (1 in)	Classification	*n*	Prev.	Rate (1 in)
V-pattern exo-deviation	262	0.1322%	757	Pattern deviation	274	0.14%	723
Y-pattern deviation	6	0.0030%	33,025				
V-pattern eso-deviation	4	0.0020%	49,537				
X-pattern exo-deviation	2	0.0010%	99,074				
Superior oblique and superior rectus muscle palsies	58	0.0293%	3417	Paretic incomitance	128	0.06%	1548
Superior oblique muscle palsy	42	0.0212%	4718				
Superior rectus muscle weakness	15	0.0076%	13,210				
Inferior oblique muscle overaction and superior oblique muscle palsy	6	0.0030%	33,025				
Inferior oblique muscle overaction and superior rectus weakness	5	0.0025%	39,630				
Superior oblique muscle underaction and inferior rectus underaction	2	0.0010%	99,074				
Inferior oblique muscle overaction	106	0.0535%	1870	IO overaction	106	0.05%	1869
Brown’s syndrome	49	0.0247%	4044	Mechanical incomitance	81	0.04%	2446
Duane’s syndrome	32	0.0161%	6193				
Other	15	0.0076%	13,210	Miscellaneous	20	0.01%	9907
Miscellaneous vertical	5	0.0025%	39,630				
Nystagmus	10	0.0050%	19,815	Nystagmus			
Ptosis	7	0.0035%	28,307	Ptosis			
Missing data	6	0.0030%	33,025	Inconclusive			

#### Prevalence of Binocular Vision Anomalies (BVAnom)

Using the above definitions, an overarching classification of clinically significant BVAnom was created of all cases with any of the following: strabismus (including intermittent), vertical heterophoria, any esophoria, clinically significant exophoria and/or abnormality in the OM field. The prevalences of BVAnom for each year studied, consecutively, were as follows: 3.02, 3.78, 3.83, 4.87, 4.89% (*r*^2^ = 0.930, *p* = 0.008; Fig. 1).

### Second Aim: Adequacy of Vision Screening That Assesses Presenting Vision Alone

For each year in the dataset, the data were analysed to determine the proportion of cases with a BVAnom who did not meet the presenting vision criteria [21] for failing vision screening. The results (Table 4) reveal a statistically significant increase in prevalence over time (*r*^2^ = 0.934, *p* = 0.007).

**Table 4:** Cases (in the eight HBs that returned data for every year analysed) with a BVAnom that would have passed vision screening based on presenting vision criteria alone, presented as absolute number, proportion of those screened (prevalence) and proportion of those with BVAnom.

	2013–2014	2014–2015	2015–2016	2020–2021	2021–2022
Number Prevalence Proportion of BVAnom	342 0.81% 27.0%	372 0.88% 23.2%	400 0.97% 25.2%	584 1.59% 32.7%	512 1.44% 29.4%

*BVAnom* binocular vision anomalies, *HBs* Health Boards.

## Discussion

### Summary of Main Findings and Interpretation

The cover test data reveal a statistically significant increase in the prevalence of exotropia from 2013–2022. Previously reported findings from this dataset show increasing myopia, even at this young age of 3.5–5.5 years [22]. Myopia is more often associated with exo-deviations relative to hyperopia and eso-deviations[18], and the present results confirm this relationship (Fig. 2), so this trend is not surprising. However, the present data reveal no statistically significant decrease in esotropia over time. For all strabismus, there is a statistically significant increase in prevalence over the period studied.

Acute acquired concomitant esotropia (AACE) is typically associated with myopia [27] (c.f., accommodative esotropia), and has increased in prevalence since the pandemic [28]. However, AACE tends to have an older age of onset than studied here [27, 28], making it an unlikely explanation for the stable esotropia prevalence in the present cohort.

The prevalence of an abnormal OM result is relatively stable over time. Pattern deviation was the most commonly recorded category as a comment by screener orthoptists, followed by incomitances such as superior oblique and superior rectus muscle palsies, inferior oblique overaction, Brown’s syndrome, superior oblique muscle palsies and Duane’s syndrome. Since the purpose of screening is to detect abnormalities rather than to diagnose, not all cases where OM was coded as abnormal included a comment describing the OM anomaly. This, together with low numbers of these rare conditions each year, is why annual prevalences have not been calculated for these individual conditions. However, it is noteworthy that the prevalence of an abnormal result at the OM test each year is approximately 0.5–1.0%.

To address the first aim, when abnormal cover test and OM test results are considered together, the overall prevalence of BVAnom ranges from approximately 3–5%, with a statistically significant increasing trend over time (Fig. 1). For both of the most recent 2 years, the prevalence of BVAnom was 4.9%.

Concerning the second aim, the prevalence of BVAnom that would not have been detected by solely applying the fail criteria relating to presenting vision was 0.81–0.97% in the first 3 years of data, increasing to ~1.5% in the last 2 years (Table 4). This supports the value of having screening conducted by orthoptists and including basic tests of binocular function. The ‘value added’ by using orthoptists is increasing over time, which is not surprising in view of the increasing prevalence of BVAnom. Earlier analyses of this cohort reveal an increasing prevalence of myopia [22], also found globally [29], and as noted above, this increases the risk of BVAnom [18]. Other potential reasons for increasing BVAnom include the increased survival rates of premature infants.

This finding highlights the limitations of vision screening programmes that only assess presenting monocular vision [30], often tested by school nurses rather than eye care professionals [30]. Additionally, screening instruments that assess refractive errors [31–33] are unlikely to identify a significant proportion of the BVAnom detected in screening programmes that include cover testing and OM testing.

### Comparison with the Literature

Recently, reports on large databases of child vision screening data in England [34] and Scotland [21] describe worsening presenting vision. The present team’s previous publication on the See4School data reveals that it is unsafe to assume that children with uncorrected or under-corrected myopia will self-refer [22]. The present data indicate that many children will also have BVAnom that would not have been detected had it not been for vision screening.

The increasing prevalence of strabismus in the present data is concerning. Strabismus, in addition to being an important risk factor for amblyopia [18], interferes with employment [14], motor skills [35], reading speed [36], dating [13] and quality of life [12, 37]. A recent review also highlighted the value of vision screening for strabismus [38].

The prevalence of strabismus of 2.0% and 2.2% in the last 2 years of data is similar to the 1.6% found in Danish children [39] and agrees with childhood strabismus prevalence on the UK Royal College of Ophthalmologists website of 2.1% [40]. A systematic review and meta-analysis found strabismus prevalence varied markedly in different countries and over time [23]. This meta-analysis revealed a pooled prevalence of exotropia of 1.23% (95% CI: 1.00–1.46), esotropia of 0.77% (0.59–0.95) and strabismus in total of 1.93% (1.64–2.21). Like the present study, there was an increasing prevalence of exotropia over time. There is controversy over the prevalence of decompensated heterophoria owing to differences in diagnostic criteria [18]. Typically, studies of BVAnom prevalence in children provide minimal information about OM anomalies [39, 41], probably owing to the rarity of these conditions compared with strabismus and heterophoria [18].

Garretty considers it is difficult to argue that strabismus without the potential for binocular vision needs to be identified at screening because surgery at age 5–6 years may not be more beneficial than at a later date [42]. However, this reasoning is unlikely to apply to intermittent strabismus, which often can be managed non-surgically [18], with less risk of deterioration to a constant strabismus [18], and improved visual performance [43]. When strabismus surgery is required, this is cost-effective [44]. A Royal College of Ophthalmologists review concluded that strabismus surgery in adults should not be rationed on non-clinical grounds [44]. Although this referred to adults, it would seem sensible to apply the standards also to children.

Patients with intermittent distance exotropia may not complain of symptoms [18], and this raises questions about whether it is important to detect the condition. Research indicates that intermittent exotropia can affect health-related quality of life [45]. Another study found that 17% of cases required treatment for reduced visual acuity, 13% required non-surgical treatment and 16% surgery [46]. Additionally, reduced distance stereoacuity [47] and impaired contrast sensitivity [48] are common findings in patients with intermittent exotropia. Intermittent distance exotropia can respond well to both non-surgical [18] and surgical interventions [49]. Even for cases that do not require treatment, providing a diagnosis helps patients recognise symptoms that may need future care and understand how issues like diplopia or reduced stereopsis could affect daily activities. This supports informed decision-making, in accordance with National Health Service guidance [50].

There are additional reasons why the detection of BVAnom may be helpful. A recent review concludes that children with reading difficulties (e.g., dyslexia) are particularly likely to have BVAnom [51]. In secondary school children, an association has been found between BVAnom and lower scores at national examinations [52]. Also, computer vision syndrome (digital eye strain), which is increasingly common in children [53–56], is sometimes attributable to BVAnom [57–59].

McCullough and Saunders evaluated the visual profile of children who passed or failed the UK school vision screening protocol [19]. Pass/Fail criteria for the screening test were based on monocular presenting vision, and, in addition, children underwent an eye examination, including a cover test at distance and near. From the study population of 294 children, 9 (3.2%) had manifest strabismus, and of these, 2 (22.2%) passed the vision screening test (i.e., would not have been detected by vision screening that did not include a cover test). Both this and the present study confirm that a significant proportion of cases of clinically significant BVAnom would not be detected by vision screening that is based solely on presenting vision.

### Implications

At present, child vision screening in the UK has the primary goal of detecting amblyopia [1, 3]. In 2013, an external review against programme appraisal criteria asked whether the current UK screening at age 4–5 years met NSC criteria [60]. The review ‘found no robust evidence to support significant changes’, but did not consider whether a broadening of the programme was appropriate to consider other conditions. Analysis of refractive error data from the See4School programme indicates that refractive errors should be considered [22], and the present data also supports the inclusion of testing for BVAnom.

Broadening the focus of child vision screening from solely considering amblyopia to include refractive errors and BVAnom should trigger a re-evaluation of considerations such as the optimal age and repeat screening episodes, to be more in line with vision screening programmes in other countries [6, 7]. To determine the frequency of screening, the limiting condition is probably myopia [61, 62], because this is likely to have developed in over 50% of young people by the time they become university students [63]. In a European population, Polling et al. showed that the median rate of progression of myopia is approximately 0.50 D per annum up to the age of 10 years and slower thereafter [64]. Therefore, biennial screening throughout primary school (under 12 years of age) and triennial screening in secondary school (≥ 12 years of age) would seem appropriate. The clinically significant prevalence of BVAnom in the present analyses leads to the recommendation that this vision screening throughout the school years should aim to detect not only refractive errors but also BVAnom.

The present work has not evaluated cost-effectiveness. In the UK, Horwood et al. have shown that even when only refractive errors and amblyopia are considered, vision screening by orthoptists at school-entry is cost-effective compared with screening involving school nurses [4]. Baltussen et al. concluded in 2009 that vision screening for refractive errors in older children is economically attractive [65]. The increased prevalence of myopia since then is likely to make the argument for vision screening even more compelling.

### Strengths and Limitations

Strengths of the present work include the large sample size, which results in robust estimates of prevalence. Another strength is that the study population originated in community screening, rather than a clinical population. As noted in the introduction, there is a lack of good data on the prevalence of BVAnom in young children in the UK.

The inclusion of IOD in presenting vision as a fail criterion is considered a strength. This criterion was set after local audits (unpublished), and the sensitivity and specificity of the criterion have not been assessed.

The use of free-text comments for categorising OM anomalies may introduce variability, which was mitigated by authors with expertise in BVAnom categorising the comments. The See4School programme does not formally assess inter-rater reliability or consistency. However, the British and Irish Orthoptic Society conducts an annual vision screening audit to ensure consistent procedures across all regions. In the 2020–2021 audit, Scotland achieved a 64.3% response rate, with all sites (100%) compliant with NHS Scotland guidelines [66]. The audit enhances data quality and consistency by reducing errors and ensuring accurate identification of children requiring further assessment. All screeners are registered orthoptists who work in HES teams, which routinely cross-check each other, and who are randomly audited by the Health and Care Professions Council.

As a result of the delay in screening in the year of the pandemic (2020–2021), the mean age of participants at the time of screening is likely to be slightly older in that year than in other years [22]. Date of screening and age are not stored in the database available for analysis, and therefore, this delay cannot be quantified.

The cover test data comprised information on the type of deviation (Table 1). Data on the magnitude of deviations were not recorded. Similarly, a full battery of tests for diagnosing decompensated heterophoria [18] is not appropriate in the screening setting. Nonetheless, a strength is that the screening was undertaken by orthoptists, so the cover test and OM data are likely to be reliable.

Stereopsis testing in the screening was a simple present/absent criterion based on the first plate of the Frisby stereo test or gross plates of the TNO test. Previous research has found that results with different editions of printed stereo tests are not comparable [67] and that, depending on the test, 9–29% of 3-year-old children cannot be tested with stereo tests [68].

Many vision screening programmes do not include a pass/fail criterion based on the IOD in presenting vision, and the criterion in the See4School programme of three letters or more [20] is particularly sensitive. This is likely to have increased the sensitivity of the programme’s presenting vision testing for detecting BVAnom, and, therefore, the present analyses may have underestimated the extent to which orthoptic testing improves the ability to detect BVAnom.

Another limitation is that data for 2016–2020 are not available. No assumptions were made in the analyses regarding missing data, which is a conservative approach. Data imputation [69] was considered, but this requires assumptions that may be incorrect, leading to erroneous conclusions [70]. Whilst there is no reason to consider that the trend in the years for which data were missing would differ markedly from the preceding years, the absence of data means that this cannot be inferred with certainty. A final limitation is that no data are available on false negatives.

## Conclusions

In a large population of mainly Caucasian children in Scotland aged 3.5–5.5 years, there is an increasing prevalence of BVAnom, especially exotropia. Many cases of BVAnom would not be detected by solely assessing presenting vision, highlighting the benefits of including a wider range of tests undertaken by eye care professionals.

## Data Availability

The dataset will be made available following any reasonable request to the authors.
